# Impact of COVID-19 on liver function: results from an internal medicine unit in Northern Italy

**DOI:** 10.1007/s11739-020-02425-w

**Published:** 2020-07-10

**Authors:** Marco Vincenzo Lenti, Federica Borrelli de Andreis, Ivan Pellegrino, Catherine Klersy, Stefania Merli, Emanuela Miceli, Nicola Aronico, Caterina Mengoli, Michele Di Stefano, Sara Cococcia, Giovanni Santacroce, Simone Soriano, Federica Melazzini, Mariangela Delliponti, Fausto Baldanti, Antonio Triarico, Gino Roberto Corazza, Massimo Pinzani, Antonio Di Sabatino, the Internal Medicine Covid-19 Team, Gaetano Bergamaschi, Giampiera Bertolino, Silvia Codega, Filippo Costanzo, Roberto Cresci, Giuseppe Derosa, Francesco Falaschi, Carmine Iadarola, Elisabetta Lovati, Pietro Carlo Lucotti, Alessandra Martignoni, Amedeo Mugellini, Chiara Muggia, Patrizia Noris, Elisabetta Pagani, Ilaria Palumbo, Alessandro Pecci, Tiziano Perrone, Carla Pieresca, Paola Stefania Preti, Mariaconcetta Russo, Carmelo Sgarlata, Luisa Siciliani, Andrea Staniscia, Francesca Torello Vjera, Giovanna Achilli, Andrea Agostinelli, Valentina Antoci, Alessia Ballesio, Francesco Banfi, Chiara Barteselli, Irene Benedetti, Michele Brattoli, Francesca Calabretta, Ginevra Cambiè, Roberta Canta, Federico Conca, Luigi Coppola, Elisa Maria Cremonte, Gabriele Croce, Virginia Del Rio, Francesco Di Terlizzi, Maria Giovanna Ferrari, Sara Ferrari, Anna Fiengo, Tommaso Forni, Giulia Freddi, Chiara Frigerio, Federica Fumoso, Alessandra Fusco, Margherita Gabba, Matteo Garolfi, Antonella Gentile, Giulia Gori, Giacomo Grandi, Paolo Grimaldi, Alice Lampugnani, Francesco Lapia, Federica Lepore, Gianluca Lettieri, Jacopo Mambella, Chiara Mercanti, Francesco Mordà, Alba Nardone, Luca Pace, Lucia Padovini, Alessandro Parodi, Lavinia Pitotti, Margherita Reduzzi, Giovanni Rigano, Giorgio Rotola, Umberto Sabatini, Lucia Salvi, Giovanni Santacroce, Jessica Savioli, Simone Soriano, Carmine Spataro, Debora Stefani

**Affiliations:** 1 Department of Internal Medicine, San Matteo Hospital Foundation, University of Pavia, Pavia, Italy; 2 Biometry and Clinical Epidemiology Service, San Matteo Hospital Foundation, Pavia, Italy; 3 Molecular Virology Unit, Microbiology and Virology Department, San Matteo Hospital Foundation, Pavia, Italy; 4 Chief Medical Direction, San Matteo Hospital Foundation, Pavia, Italy; 5 UCL Institute for Liver and Digestive Health and Sheila Sherlock Liver Unit, Royal Free Hospital and UCL, London, UK; 6 Clinica Medica, Fondazione IRCCS Policlinico San Matteo, Università di Pavia, Viale Golgi 19, 27100 Pavia, Italy

**Keywords:** Acute respiratory failure, Coronavirus, Hepatitis, Severe acute respiratory syndrome

## Abstract

Little is known regarding coronavirus disease 2019 (COVID-19) clinical spectrum in non-Asian populations. We herein describe the impact of COVID-19 on liver function in 100 COVID-19 consecutive patients (median age 70 years, range 25–97; 79 males) who were admitted to our internal medicine unit in March 2020. We retrospectively assessed liver function tests, taking into account demographic characteristics and clinical outcome. A patient was considered as having liver injury when alanine aminotransferase (ALT) was > 50 mU/ml, gamma-glutamyl transpeptidase (GGT) > 50 mU/ml, or total bilirubin > 1.1 mg/dl. Spearman correlation coefficient for laboratory data and bivariable analysis for mortality and/or need for intensive care were assessed. A minority of patients (18.6%) were obese, and most patients were non- or moderate-drinkers (88.5%). Liver function tests were altered in 62.4% of patients, and improved during follow-up. None of the seven patients with known chronic liver disease had liver decompensation. Only one patient developed acute liver failure. In patients with altered liver function tests, PaO_2_/FiO_2_ < 200 was associated with greater mortality and need for intensive care (HR 2.34, 95% CI 1.07–5.11, *p* = 0.033). To conclude, a high prevalence of altered liver function tests was noticed in Italian patients with COVID-19, and this was associated with worse outcomes when developing severe acute respiratory distress syndrome.

**Electronic supplementary material:**

The online version of this article (10.1007/s11739-020-02425-w) contains supplementary material, which is available to authorized users.

## Introduction

Described for the first time in December 2019 in Wuhan (China) [1, 2], coronavirus disease 2019 (COVID-19), caused by severe acute respiratory syndrome coronavirus 2 (SARS-CoV-2, previously known as 2019-nCoV), has rapidly spread in late February 2020 in Northern Italy, and, to a lesser extent, in other parts of the country [3]. In a matter of weeks, as of 29th April 2020, the epidemic reached more than 200,000 individuals, with a high mortality rate, according to preliminary data released by the Italian Ministry of Health and the Italian Civil Protection [4]. Due to its worldwide spread, the World Health Organization declared COVID-19 a pandemic on 11th March 2020 [5].

COVID-19 pathogenesis, natural history, and optimal treatment still need to be elucidated, and little is known about its clinical spectrum in non-Asian populations. COVID-19 may be symptomless in many cases [6] and, when symptomatic, usually causes a flu-like syndrome (dry cough, sore throat, fever, diffuse muscle pain) that may be complicated by interstitial pneumonia with SARS and superimposed bacterial infections which may be fatal [2, 7–10]. In fact, similar to SARS-CoV, SARS-CoV-2 is likely to bind to the angiotensin-converting enzyme 2 receptor, which is highly expressed in the respiratory tract, hence tissue damage mostly occurs in this site [11–13]. However, gastrointestinal involvement may precede the onset of respiratory symptoms, and patients may experience abdominal pain, nausea and diarrhoea during the course of the disease [14]. Also, in a substantial proportion of patients, especially those with a more severe disease, liver impairment may occur [15]. In previous Asian series, alanine aminotransferase (ALT) and aspartate aminotransferase (AST) elevation has been reported in roughly one-third of COVID-19 patients, and even a few cases of acute liver failure were observed [9, 15–20]. The mechanisms underlying liver impairment in COVID-19 are unknown, but they may be directly caused by the virus, or indirectly via other pathways (e.g., inflammation, hypoxia, medications). Also, nothing is known about liver impairment in non-Asian patients, and its clinical impact in these patients is poorly investigated.

Starting from these premises, the aim of this study was to describe the clinical characteristics of a cohort of patients with COVID-19 admitted to an internal medicine ward of an academic, tertiary referral hospital, with particular regard to liver impairment, and in relation to the clinical outcome. We also described the impact of COVID-19 in a sub-cohort of patients affected by chronic liver disease.

## Materials and methods

In this single-centre, retrospective, observational study, we collected data from patients with Covid-19 who were admitted between 5th March and 28th March 2020 (last follow-up on 1st April 2020) to the internal medicine unit of San Matteo Hospital Foundation (Pavia, Italy), which comprises 76 beds usually occupied by patients referred from the local A&E Department. During the SARS-CoV-2 Italian epidemic, the internal medicine unit was transformed into a “COVID” ward [21] to overcome the dramatic increase of infected patients needing hospitalisation in Lombardy [22]. Demographic and clinical data of these patients were extracted and anonymised from the electronic hospital records onto a pre‐defined spreadsheet. Relevant data included sex, age, smoking status, body mass index (BMI), alcohol intake, past medical history, and clinical description of COVID-19 at onset. We included all laboratory data that were requested as per clinical need, using the local laboratory reference level of normality as cut-off, at the time of admission (first timepoint). Liver function was considered impaired when ALT > 50 mU/ml, and/or gamma-glutamyl transpeptidase (GGT) > 50 mU/ml, and total bilirubin > 1.1 mg/dl (this latter when associated with altered transaminases and with no other causes of increase). Other relevant laboratory liver tests included serum cholinesterase, alkaline phosphatase (ALP), serum albumin, international normalised ratio (INR), serum glucose, and urine ketones. Lactate dehydrogenase (LDH) was assessed as an unspecific marker of cytolysis, while c reactive protein (CRP) was included as unspecific inflammatory marker. Neutrophil-to-lymphocyte ratio was included as a marker of stress used in critically ill patients [23]. Arterial oxygen partial pressure to fractional inspired oxygen ratio (PaO_2_/FiO_2_) was used for assessing the severity of respiratory failure. Diagnosis of COVID-19 was based on clinical grounds (flu-like symptoms, fever, tachypnoea, hypoxemia, presence of radiological interstitial pneumonia) and SARS-CoV-2 detection through nasopharyngeal swab. More in depth, total nucleic acids (DNA/RNA) were extracted from samples (200 µl) using the QIAsymphony^®^ instrument with QIAsymphony^®^ DSP Virus/Pathogen Midi Kit (Complex 400 protocol) following the manufacturer’s instructions (QIAGEN, Qiagen, Hilden, Germany). Specific real-time PCR targeting RNA-dependent RNA polymerase and E genes were used to detect the presence of SARS-CoV-2 according to internationally recognised criteria [24, 25]. In a few patients with negative nasopharyngeal swab, but high clinical suspicious, diagnosis was made through bronchoalveolar lavage. Nasopharyngeal swab for ruling out other possible viral co-infections of the upper respiratory tract (i.e., influenza A and B, parainfluenza viruses, syncytial respiratory virus, rhinoviruses, adenovirus, other coronaviruses) was also performed.

The study was performed as a clinical audit using routine collected clinical data and as such is exempt from the need to take specific written informed consent. The study was approved by the local ethics committee (San Matteo Hospital Foundation) on 13th March 2020. The results of this study are reported according to the STrengthening the Reporting of OBservational studies in Epidemiology (STROBE) recommendations [26].

### Statistical analysis

Given the observational nature of the study, sample size was not calculated a priori. A descriptive statistical analysis was performed for clinical features, and data were expressed as number of total and/or percentage, mean and standard deviation (SD), or median and range when appropriate. When variables were not available for some patients, these were excluded for percentage calculation. Comparison amongst groups at univariable analysis was performed by the Wilcoxon matched-pairs sign rank test. Spearman correlation coefficient was calculated for relevant laboratory parameters, together with its 95% confidence interval (95% CI). For all correlations, patients with a known history of chronic liver disease and those taking anticoagulants were excluded. Also, patients with a history of type 2 diabetes mellitus were excluded when considering urine ketones. Univariable and bivariable Cox regression models were fitted. Hazards ratios (HR) and 95% CI were computed. Kaplan Meier event-free survival was computed and plotted for patients who had altered liver function tests versus those who had normal ranges. For the analyses of mortality and the composite outcome mortality plus need for intensive care, the role of altered liver function was adjusted, in turn, for the following variables: age > 70 years, female sex, BMI > 25, number of comorbidities, PaO_2_/FiO_2_ < 200 (moderate-severe acute respiratory distress syndrome) [27], LDH > 450 mU/ml, and neutrophil-to-lymphocyte ratio. Two-tailed *p* values less than 0.05 were considered statistically significant. The software STATA 16 (StataCorp, College Station, TX) was used for all computations.

## Results

Over the study period, 152 patients were admitted to the internal medicine unit. Of these, 52 patients investigated for clinical suspicious for Covid-19, but with no evidence of SARS-CoV-2 in the collected biological specimens, were excluded from the study. In the remaining 100 cases (median age 70 years old, range 25–97; 79 males; 99 Caucasian and 1 Asian patient), a definite diagnosis of COVID-19 was made on the basis of a SARS-CoV-2-positive nasopharyngeal swab. In four patients, SARS-CoV-2 was detected only through bronchoalveolar lavage. Table 1 summarises the baseline demographic characteristics and lifestyle habits of the whole cohort, while Table 2 shows the most commonly associated conditions. Notably, only a minority (18.6%) were obese, and most patients were male (79.0%) and non-smoker (79.4%). More than half of the patients (65.0%) suffered from essential hypertension. Seven patients suffered from a known chronic liver disease, and their clinical details are reported in Table 3. Child–Pugh score was A6 for patient numbers one to five, while it was B7 for patient numbers six and seven. For the sake of homogeneity, these patients were excluded from later analyses. Even if this is just a small series, we noticed that among patients with chronic liver diseases, only one died, while the others had a relatively favourable outcome, and none of these patients had liver decompensation. Table 4 shows the clinical and radiologic features of the 93 remaining patients at the time of admission. Fever ≥ 38.0 °C (81.7%), cough (58.1%), and dyspnoea (49.5%) were the most common findings, while interstitial pneumonia was the most common chest radiological abnormality (65.6%). Overall, gastrointestinal symptoms were infrequent (12.9%), and only a minority of patients presented with diarrhoea (10.8%). Supplementary Table 1 shows the most relevant laboratory findings at the time of patient admission (none of the patients was treated with antiviral drugs at the time of the evaluation). According to the aforementioned inclusion criteria, liver function test alterations were found in 58/93 patients (62.4%). In roughly half of the cases, AST, ALT, and GGT were elevated, while INR was abnormal in 27.7% of patients at baseline. Also, serum albumin was decreased in 93.5% of the cases. Supplementary Table 1 reports the most relevant correlations between liver function tests and inflammatory or respiratory parameters. Of note, AST and ALT positively correlated between them and with total bilirubin and GGT, while an inverse correlation between albumin and CRP was noticed. Also, LDH positively correlated with AST, ALT, GGT, and total bilirubin. After exclusion of diabetic patients, no differences regarding the presence of urine ketones between patients with altered liver function tests (20/29 patients, 69%) versus those with no alterations (9/29 patients, 31.0%; *p* = 0.336) were noticed.

**Table 1:** Demographic and lifestyle habits of the overall cohort of COVID-19 patients

	Patients (*n* = 100)
Age (years), median (range)	70 (25–97)
Sex, *n* (%)	
Female	21 (21.0)
Male	79 (79.0)
Smoking status, *n* (%)	
No smoker/past smoker (≥ 10 years)	50/63 (79.4)
Past smoker (< 10 years)	7/63 (11.1)
Current smoker (< 20 cigarettes/day)	4/63 (6.3)
Current smoker (≥ 20 cigarettes/day)	2/63 (3.2)
Alcohol intake, *n* (%)	
No alcohol intake	29/61 (47.5)
< 3 alcohol units/day	25/61 (41.0)
≥ 3 alcohol units/day	6/61 (9.8)
Body Mass Index, *n* (%)	
< 18.5	3/59 (5.1)
18.5–24.9	28/59 (47.5)
25.0–29.9	17/59 (28.8)
30.0–34.9	11/59 (18.6)
≥ 35.0	0/59 (0.0)

**Table 2:** Associated conditions in the overall cohort of Covid-19 patients

	Patients (*n* = 100)
At least one associated condition, *n* (%)	84 (84.0)
Chronic ischemic heart disease	21 (21.0)
Arrhythmia	20 (20.0)
Valvular heart disease	11 (11.0)
Essential hypertension	65 (65.0)
Atherosclerosis	20 (20.0)
Chronic obstructive pulmonary disease	7 (7.0)
Asthma	6 (6.0)
Chronic interstitial pneumonitis	2 (2.0)
Type 2 diabetes mellitus	16 (16.0)
Treatment	
Diet	3 (3.0)
Metformin	6 (6.0)
DPP4-inhibitor (sitagliptin, linagliptin)	1 (1.0)
Other oral hypoglycaemic agents	2 (2.0)
Insulin	7 (7.0)
Chronic liver disease	7 (7.0)
Non-alcoholic fatty liver disease	2 (3.0)
HBV-related	2 (2.0)
HCV-related	1 (1.0)
Hemochromatosis	1 (1.0)
Alcohol-related	1 (1.0)
Thyroid disease	9 (9.0)
Hyperthyroidism	2 (2.0)
Hypothyroidism	7 (7.0)
Chronic kidney disease	13 (13.0)
Onco-hematologic disease	6 (6.0)
Neoplastic history	13 (13.0)
Previous history of neoplastic disease	4 (4.0)
Active neoplastic disease	9 (9.0)
Autoimmune disease (any)	10 (10.0)
Connective tissue disease	6 (6.0)

DPP4, dipeptidyl peptidase-4; HBV, hepatitis B virus; HCV, hepatitis C virus

**Table 3:** Characteristics of the seven COVID-19 patients with a known history of chronic liver disease

Patient	Age	Sex	Chronic liver disease	Other associated conditions	Clinical presentation	Liver function tests at baseline and follow-up	Clinical outcome	Length of stay
1	75	M	Hemochromatosis	Thalassemia minor	Fever Interstitial pneumonia	AST (mU/ml): 62 $\to$ 34 ALT (mU/ml): 36 $\to$ 20 GGT (mU/ml): 51 $\to$ 26 Albumin (g/dl): 3.3 $\to$ 1.9 INR: 1.02 $\to$ 1.42	ICU	3
2	60	M	NAFLD	No	Cough, fever, dyspnoea, vomiting Interstitial pneumonia	AST (mU/ml): 30 $\to$ 27 ALT (mU/ml): 21 $\to$ 30 GGT (mU/ml): 60 $\to$ 90 Albumin (g/dl): 2.8 $\to$ 3.0 INR: 1.23 $\to$ 1.2	ICU	2
3	77	F	HCV-related	Advanced ovarian cancer	Cough, fever, dyspnoea Bilateral chest consolidations	AST (mU/ml): 14 $\to$ 20 ALT (mU/ml): 9 $\to$ 12 GGT (mU/ml): 43 $\to$ 42 Albumin (g/dl): 3.5 $\to$ 3.5 INR: 1.11 $\to$ 1.32	Deceased	5
4	68	M	HBV-related	Valvular heart disease, essential hypertension, chronic kidney disease, atherosclerosis	Cough, fever, and progressive dyspnoea Interstitial pneumonia	AST (mU/ml): 16 $\to$ 11 ALT (mU/ml): 11 $\to$ 21 GGT (mU/ml): 29 $\to$ 48 Albumin (g/dl): 3.6 $\to$ 3.2 INR: 1.11 $\to$ 1.52	Still hospitalised	14
5	51	M	NAFLD	Essential hypertension, atherosclerosis, type 2 diabetes mellitus	Cough, fever Interstitial pneumonia	AST (mU/ml): 49 $\to$ 41 ALT (mU/ml): 35 $\to$ 39 GGT (mU/ml): 181 $\to$ 138 Albumin (g/dl): 3.3 $\to$ 3.2 INR: 1.21 $\to$ 1.16	Discharged	11
6	73	M	Alcohol-related	Essential hypertension, type 2 diabetes mellitus, chronic kidney disease	Asymptomatic Normal chest radiological findings	AST (mU/ml): 108 $\to$ 96 ALT (mU/ml): 29 $\to$ 25 GGT (mU/ml): 113 $\to$ 112 Albumin (g/dl): 1.8 $\to$ 2.5 INR: 1.65 $\to$ 1.4	Discharged	7
7	82	M	NAFLD	Ischemic heart disease, essential hypertension, type 2 diabetes mellitus, chronic kidney disease	Cough, fever Normal chest radiological findings	AST (mU/ml): 16 $\to$ 10 ALT (mU/ml): 9 $\to$ 9 GGT (mU/ml): 6 $\to$ 11 Albumin (g/dl): 2.7 $\to$ 2.8 INR: 0.94 $\to$ 0.99	Still hospitalised	8

ALT, alanine aminotransferase; AST, aspartate aminotransferase; GGT, gamma-glutamyl transpeptidase; INR, international normalized ratio; NAFLD, non-alcoholic fatty liver disease; ICU, intensive care unit

**Table 4:** Clinical features of the 93 Covid-19 patients, who entered into the study, at the time of hospital admission

	Patients (*n* = 93)
Fever, *n* (%)	
< 38.0 °C	5 (5.4)
≥ 38.0 °C	76 (81.7)
Cough, *n* (%)	54 (58.1)
Dyspnoea, *n* (%)	46 (49.5)
Gastrointestinal symptoms, *n* (%)	
Nausea and/or vomiting	2 (2.2)
Diarrhoea	10 (10.8)
Severe abdominal pain	1 (1.1)
Chest radiological findings, *n* (%)	
Normal	3 (3.2)
Unilateral lung consolidations	12 (12.9)
Bilateral lung consolidations	17 (18.3)
Interstitial pneumonia	61 (65.6)

**Fig. 1:**
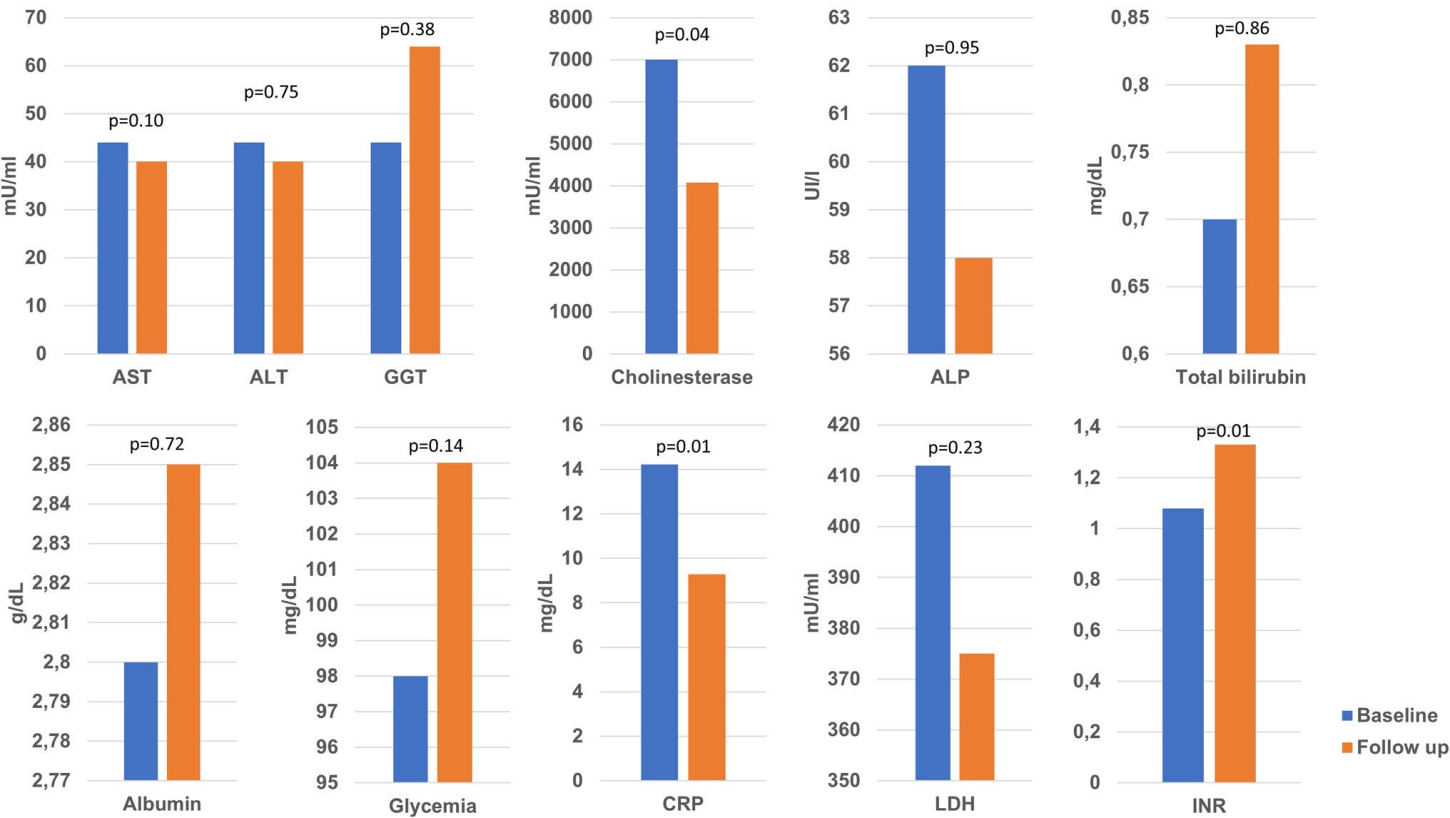
Comparison of medians of blood tests indicative of abnormal liver function at hospital admission (baseline) and at last follow-up. Wilcoxon test was performed to evaluate statistical significance. ALT, alanine aminotransferase; ALP, alkaline phosphatase; AST, aspartate aminotransferase; CRP, C reactive protein; GGT, gamma-glutamyl transpeptidase; INR, international normalised ratio; LDH, lactate dehydrogenase

As shown in Fig. 1, at the last follow-up during hospitalisation (median of 8 days after admission), AST and ALT showed a decreasing trend, while GGT and bilirubin showed an increasing trend, but none of these variations were statistically significant compared to baseline. Instead, INR showed a statistically significant increasing trend at the last follow-up (*p* < 0.01; Fig. 1). Only one male patient aged 72 years, who was not treated with antiviral medications and who was being treated with hydroxychloroquine and antibiotics, developed acute liver failure concomitant to acute respiratory distress syndrome (peak ALT 70 IU/l, AST 143 IU/l, GGT 162 IU/l, total bilirubin 15.6 mg/dl, conjugated bilirubin 11.7 mg/dl, INR 1.51, and PaO_2_/FiO_2_ 87) and was transferred to the intensive care unit. In addition, one female patient aged 58 years with a transplanted liver had a very mild disease course (no signs of interstitial pneumonia, or liver function test abnormalities).

Regarding clinical outcomes for the whole cohort, at the time of the writing of the paper, these were available in 71/100 patients (71.0%), while 29 patients were still hospitalised, and were considered as censored for the purpose of the analysis. The mean length of stay was 7 ± 4 days, 17 (23.9%) were discharged to home care, 15 (21.1%) improved and were transferred to a subacute care unit, 14 (19.7%) worsened and were transferred to the intensive care unit, and 25 (35.2%) died in hospital. Supplementary Fig. 1 shows the unadjusted Kaplan Meier survival estimate and the unadjusted Kaplan Meier event-free survival estimate (death or need for intensive care); no differences were noticed between patients who had liver function test alterations and the others. Regarding mortality only in patients with liver function test alterations versus absence of alterations, no difference was seen between the two groups at bivariable analysis when adjusted for age > 70 years (HR 1.74, 95% CI 0.73–4.15, *p* = 0.210), female sex (HR 0.93, 95% CI 0.34–2.54, *p* = 0.884), BMI > 25 (HR 0.47, 95% CI 0.14–1.52, *p* = 0.207), number of comorbidities (HR 1.74, 95% CI 0.75–4.04, *p* = 0.194), PaO_2_/FiO_2_ < 200 (HR 1.72, 95% CI 0.69–4.25, *p* = 0.241), LDH > 450 (HR 0.83, 95% CI 0.33–2.09, *p* = 0.696), and neutrophil-to-lymphocyte ratio (HR 0.87, 95% CI 0.38–2.03, *p* = 0.754). Regarding the combined outcome death or need for intensive care, only PaO_2_/FiO_2_ < 200 (HR 2.34, 95% CI 1.07–5.11, *p* = 0.033) was significantly associated with patients who had altered liver function tests, while the other variables had to statistical significance (data not shown).

## Discussion

We herein described the first Italian, non-Asian, series of patients suffering from COVID-19, admitted to an internal medicine ward, focusing on their clinical picture and liver function impairment. We found that most patients were male, older adults, non-smokers, and non-drinkers. Fever, cough, and dyspnoea were the most common symptoms, while gastrointestinal manifestations were infrequent. After excluding the few cases with a known chronic liver disease, we found that half of the patients presented with raised ALT, AST, or GGT levels. We also observed a single case of acute liver failure in the context of an acute respiratory distress syndrome. At last follow-up, liver function tests improved in most cases, but patients who had altered liver function tests, when adjusted for moderate–severe acute respiratory distress syndrome (PaO_2_/FiO_2_ < 200), showed higher mortality and need for intensive care.

This study has indeed some limitations. First, we did not provide a causal effect between COVID-19 and abnormal liver function tests, as liver specimens were not obtained from these patients. Hence, a direct cytopathic effect caused by the virus could not be ascertained, even if, according to animal models and small case series, other respiratory tract viruses were shown to have a direct cytopathic effect in the liver [28, 29]. Given the retrospective, real-life nature of the study, we could not establish whether other confounding factors played a role (e.g., previous intake of medications, other undiagnosed liver diseases). Also, the sample size of the study is rather small, thus the generalisability of our results is limited. Nonetheless, we have herein presented the clinical picture, laboratory data, and health outcomes of a cohort of mostly Caucasian patients suffering from COVID-19, focusing on liver function alterations. Also, a major strength of our series, is that only few patients suffered from overt obesity, and only few patients were active alcohol drinkers and smokers. This setting gave us the opportunity to report data regarding liver impairment in patients in whom the prevalence of unhealthy lifestyle habits was very low.

The baseline characteristics and outcomes of a large cohort of Italian patients admitted to the intensive care units in Lombardy have recently been described [30]. Our setting—that of internal medicine—provides a totally different picture of the disease, as patients admitted here had a moderate-to-severe disease, with no need for mechanic intubation at baseline, but who were also treated with continuous positive airway pressure in some cases. Hence, a wide variety of patients, in terms of age, sex, comorbidities, and COVID-19 severity have been included. Most patients were referred not only from the local A&E Department, but also from other hospital specialty wards not dedicated to the treatment of COVID-19, and patients referred to our unit were neither too compromised to need immediate access to intensive care unit nor had a mild disease not requiring hospitalisation.

According to preliminary data, the expression level and expression pattern of the ACE2 gene, encoding for angiotensin-converting enzyme-2, are different between East Asian and other populations, and this may justify a different susceptibility or response to COVID-19 in patients of different ethnicities [31]. East Asian populations have a higher expression of ACE2, and a few cases of acute liver failure have been reported [9, 15–20]. In our series, only one patient, with no history of chronic liver disease developed acute liver failure, but in this case, it was associated with severe respiratory failure. Also, data regarding COVID-19 in liver transplanted patients are scant, and we have here reported the case of one patient who had a very mild disease with no liver involvement.

The impact of COVID-19 in patients with chronic liver disease is still largely unknown. In our series, only few had a history of liver disease, and we did not notice a worse outcome in these patients, or liver decompensation. However, the small sample size does not allow us to draw any firm conclusion. We can assume that liver function test alterations in our patients were actually associated with the acute COVID-19 disease, rather than a pre-existing liver condition, especially considering the low prevalence of obesity and alcohol drinkers. Indeed, COVID-19-induced pneumonia certainly caused hypoxia and inflammation (“reactive” hepatitis, as described in a previous series of patients with SARS) [32], that may justify, at least partially, the biochemical alterations. This assumption is supported by the evidence that a positive correlation between LDH and transaminases was noticed, as well as a negative correlation between cholinesterase and PaO_2_/FiO_2_, and CRP and albumin. At last follow-up, only INR showed an increasing trend, and this might be due to different reasons, including prolonged fasting, prolonged active inflammation, and use of medications, including antibiotics.

Most patients included in our series suffered from multimorbidity. In turn, multimorbidity is a well-established risk factor for polypharmacy [33, 34], and this might be responsible for increased liver toxicity due to drug interactions with COVID-19-related treatments. Zhang et al. have already reported that liver impairment in COVID-19 patients could be drug related [15]. Moreover, a number of drugs that have been used for the treatment of COVID-19 and its related complications, such as hydroxychloroquine, antiviral drugs, and antibiotics, can be likely responsible for hepatotoxicity [19]. Finally, some of our patients suffered from non-alcoholic fatty liver disease that can sensitize the liver for hepatotoxicants, such as acetaminophen, but we excluded them from the final analyses.

To conclude, from a clinical point of view, our data certainly underlie the importance of monitoring liver function in hospitalised COVID-19 patients, not only for the possible risk of developing acute liver failure (1% in our series) and its implications (e.g., limit the use of potentially toxic medications, strict patient monitoring), but also for stratifying patients who could benefit from early access to the intensive care unit. This is crucial, considering that the pressure put by COVID-19 epidemic in Italy has almost led the healthcare system to collapse [3], and a fair allocation and utilisation of medical resources is a compelling need [35–37]. Considering our results, in an internal medicine setting, we would suggest monitoring liver function tests in all COVID-19 patients, being aware that in case of worsening of respiratory function, they could benefit from early transfer to the intensive care unit, before the onset of a manifest moderate-severe acute respiratory distress syndrome (PaO_2_/FiO_2_ < 200). We, therefore, envisage that future, prospective studies will focus on the impact of liver impairment in patients with COVID-19 to strengthen our results, and to further explore the outcomes of patients with chronic liver diseases.

## Electronic supplementary material

Below is the link to the electronic supplementary material.

Supplementary file1 (DOCX 23 kb)

Supplementary Figure 1. Unadjusted Kaplan Meier survival estimate (left side) and unadjusted Kaplan Meier event-free survival estimate (death or need for intensive care; right side) according to the presence/absence of liver function test alterations. Abbreviation: LFT, liver function test (PPTX 131 kb)

## Abbreviations

ALP: Alkaline phosphatase
ALT: Alanine aminotransferase
AST: Aspartate aminotransferase
BMI: Body mass index
CI: Confidence interval
COVID-19: Coronavirus disease 2019
CRP: C reactive protein
GGT: Gamma-glutamyl transpeptidase
INR: International normalised ratio
LDH: Lactate dehydrogenase
SD: Standard deviation
SARS-CoV-2: Severe acute respiratory syndrome coronavirus 2
STROBE: STrengthening the Reporting of OBservational studies in Epidemiology
