# Supplementary material for: Impact of COVID-19 on liver function: results from an internal medicine unit in Northern Italy
Source: Intern Emerg Med. 2020 Jul 10;15(8):1399–407. doi: 10.1007/s11739-020-02425-w (PMC7348571; doi:10.1007/s11739-020-02425-w)
Supplement: Supplementary file 1 — Supplementary file1 (DOCX 23 kb) [file 11739_2020_2425_MOESM1_ESM.docx]

**Supplementary Table 1.** Laboratory results of the 93 Covid-19 patients, who entered into the study, at the time of hospital admission.

| **Variables** | **Patients**  **(n=93)** |
| --- | --- |
| **Haemoglobin (g/dl), median (range)** | 12.7 (3.4-17.7) |
| Below normality range, n (%) | 41/90 (45.6) |
| **Leukocytes (n/ul), median (range)** | 6720 (700-91280) |
| Above normality range, n (%) | 22/90 (24.4) |
| Below normality range, n (%) | 13/90 (14.4) |
| **Lymphocytes (n/ul), median (range)** | 740 (120-86210) |
| Above normality range, n (%) | 4/90 (4.4) |
| Below normality range, n (%) | 79/90 (87.8) |
| **Neutrophil/lymphocyte ratio, median (range)** | 8.0 (0.1-71.2) |
| Above normality range, n (%) | 74/90 (82.2) |
| **Platelets (x10^3^/ul), median (range)** | 193 (43-573) |
| Above normality range, n (%) | 2/90 (2.2) |
| Below normality range, n (%) | 28/90 (31.1) |
| **LDH (mU/ml), median (range)** | 412 (69-886) |
| Above normality range, n (%) | 76/85 (89.4) |
| **C reactive protein (mg/dl), median (range)** | 14.2 (0.17-44.5) |
| Above normality range, n (%) | 90/90 (100.0) |
| **Procalcitonin (ng/ml), median (range)** | 0.2 (0.0-206.0) |
| Above normality range, n (%) | 30/76 (39.5) |
| **ALT (mU/ml), median (range)** | 35 (9-250) |
| Above normality range, n (%) | 43/87 (49.4) |
| **AST (mU/ml), median (range)** | 44 (9-490) |
| Above normality range, n (%) | 54/85 (63.5) |
| **GGT (mU/ml), median (range)** | 44 (6-367) |
| Above normality range, n (%) | 38/85 (44.7) |
| **ALP (Ul/l), median (range)** | 62 (24-319) |
| Above normality range, n (%) | 6/83 (7.2) |
| **Total bilirubin (mg/dl), median (range)** | 0.8 (0.2-2.6) |
| Above normality range, n (%) | 23/88 (26.1) |
| **Serum albumin (g/dl), median (range)** | 2.9 (1.8-4.5) |
| Below normality range, n (%) | 72/77 (93.5) |
| **Serum cholinesterase (mU/ml), median (range)** | 7000 (2716-14271) |
| Below normality range, n (%) | 23/81 (28.4) |
| **INR, median (range)** | 1.1 (1.0-8.8) |
| Above normality range, n (%) | 26/94 (27.7) |
| **Fasting serum glucose (mg/dl), median (range)** | 98 (66-247) |
| Below normality range, n (%) | 6/86 (7.0) |
| Above normality range, n (%) | 35/86 (40.7) |
| **Urine ketones (mg/dl)** |  |
| Presence, n (%) | 29/54 (53.7) |
| **Creatinine (mg/dl), median (range)** | 1.0 (0.4-11.2) |
| Above normality range, n (%) | 40/90 (44.4) |
| **PaO_2_/FiO_2_, median (range)** | 247 (57-430) |
| Below normality range, n (%) | 56/59 (94.9) |
| **Lactates (mmol/l), median (range)** | 1.3 (0.6-15.0) |
| Above normality range, n (%) | 15/66 (22.7) |

Abbreviations: ALP, alkaline phosphatase; ALT, alanine aminotransferase; AST, aspartate aminotransferase; GGT, gamma-glutamyl transpeptidase; INR, international normalised ratio; LDH, lactate dehydrogenase; PaO2/FiO2, arterial oxygen partial pressure to fractional inspired oxygen ratio.
