# Supplementary material for: Impact of COVID-19 on liver function: results from an internal medicine unit in Northern Italy
Source: Intern Emerg Med. 2020 Jul 10;15(8):1399–407. doi: 10.1007/s11739-020-02425-w (PMC7348571; doi:10.1007/s11739-020-02425-w)
Supplement: Supplementary file 2 — Supplementary Figure 1. Unadjusted Kaplan Meier survival estimate (left side) and unadjusted Kaplan Meier event-free survival estimate (death or need for intensive care; right side) according to the presence/absence of liver function test alterations. Abbreviation: LFT, liver function test (PPTX 131 kb) [file 11739_2020_2425_MOESM2_ESM.pptx]

## Slide 1
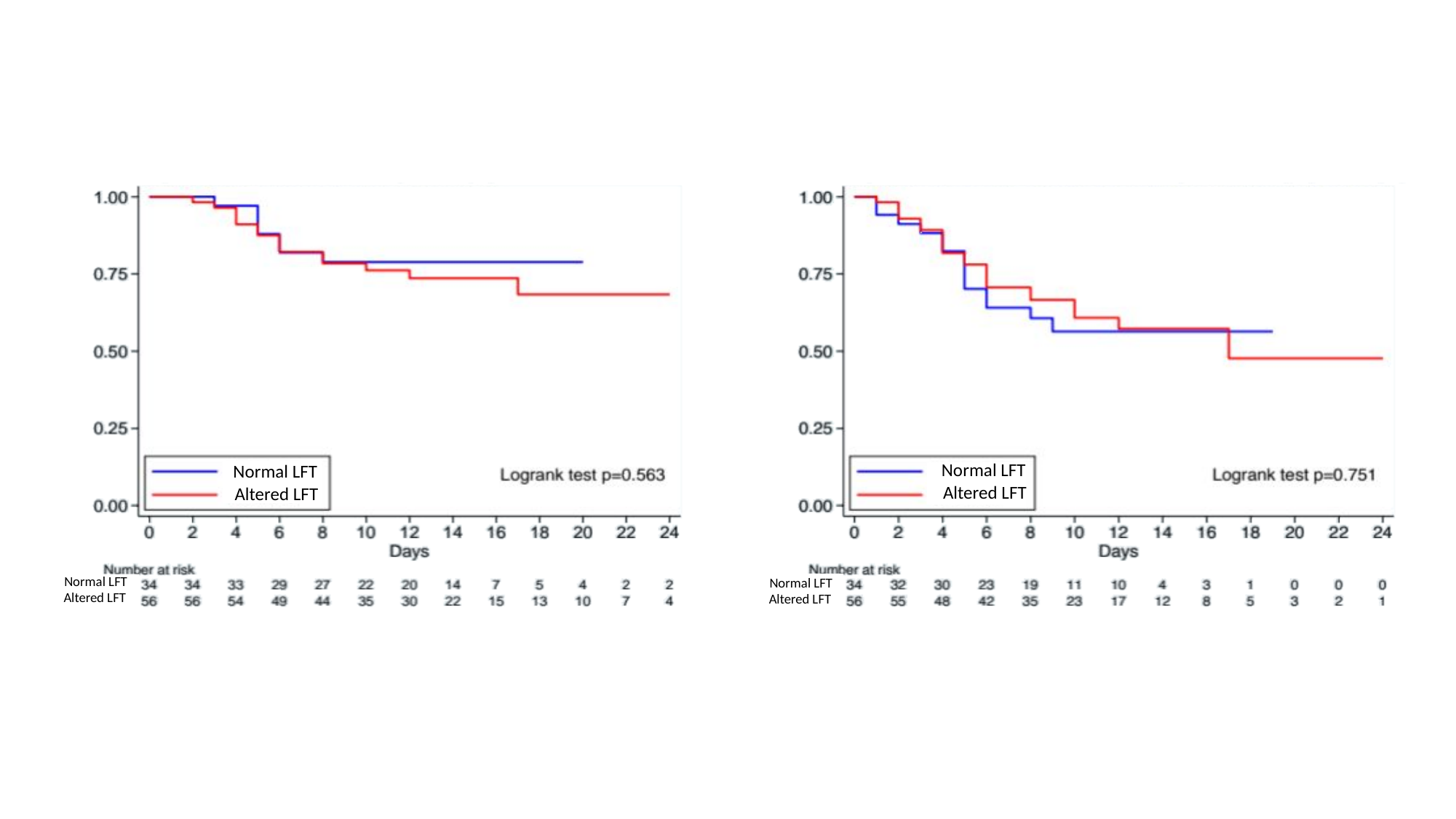

Normal LFT
Normal LFT
Altered LFT
Altered LFT
Normal LFT
Normal LFT
Altered LFT
Altered LFT
